# The Penn Vet Working Dog Center Fit to Work Program: A Formalized Method for Assessing and Developing Foundational Canine Physical Fitness

**DOI:** 10.3389/fvets.2020.00470

**Published:** 2020-08-13

**Authors:** Brian D. Farr, Meghan T. Ramos, Cynthia M. Otto

**Affiliations:** ^1^Army Medical Department Student Detachment, 187th Medical Battalion, Medical Professional Training Brigade, Joint Base San Antonio - Fort Sam Houston, San Antonio, TX, United States; ^2^Penn Vet Working Dog Center, Clinical Sciences and Advanced Medicine, School of Veterinary Medicine, University of Pennsylvania, Philadelphia, PA, United States

**Keywords:** canine physical fitness, fitness assessment, exercise program, musculoskeletal system, working dogs, performance

## Abstract

Fit to Work is a formalized working dog foundational physical fitness assessment and development program. The Penn Vet Working Dog Center developed this program to address the needs of working dog handlers, trainers, and programs for simple, effective, and efficient methods to develop and assess working dog physical fitness. Fit to Work focuses on the foundational fitness modalities of strength, stability, mobility, and proprioception. The Penn Vet Working Dog Center piloted and refined this program over 3 months in a closed population of 31 working dogs in training. Fit to Work consists of posture development and maintenance, warm-up and cool-down routines, training exercises, and assessment methods. To simplify implementation for dogs and personnel, the foundational training program incorporates a discrete number of exercises, standardized progression steps, defined criteria for progression, and a reduced emphasis on learned behaviors. Fit to Work also enables safe and progressive assessment of foundational fitness through a tiered and inexpensive process. Future research will focus on validation of training and assessment methods, development of assessment standards, and correlation of physical fitness with operational performance.

## Introduction

### Canine Physical Fitness

Canine physical fitness is a recognized requirement for safe and effective performance of working dogs. Current definitions of canine physical fitness are vague and are frequently tied to a specific type of activity (e.g., running, agility). Extrapolating from human literature, physical fitness is defined by The American College for Sports Medicine as “a set of attributes that people have or achieve” (1). The American College for Sports Medicine further defines physical fitness into measurable health and skill-related (athletic ability) attributes (1, 2). The health-related components of physical fitness are: cardiorespiratory endurance, muscular endurance, muscular strength, body composition (e.g., amount of fat vs. muscle), and mobility (1, 2). The skill-related components of physical fitness are agility, balance, coordination, speed, power, and reaction time (1). Defining canine physical fitness in these terms of measurable components enables further investigation into the details of each component and their impact on the working dog's physical requirements to perform a variety of athletic tasks. While there are other aspects of fitness important for working dogs (e.g., mental), this article will use the term “fitness” to solely refer to physical fitness.

### Foundational Fitness

Basic canine fitness requires a foundational fitness program coupled with a scientifically based and repeatable assessment process. Foundational fitness addresses mobility, stability (e.g., core, forelimb, hindlimb), strength, and proprioception required to perform basic physical tasks such as running, jumping (up and down), navigating unstable surfaces, and quickly or abruptly changing direction. The definition for each foundational fitness component can be found in Table 1. These components were selected to primarily target the muscles of the core, hind limbs, and supporting soft tissue structures (e.g., fascia, ligaments, etc.) that are not primarily engaged when a dog performs routine physical activities. Dogs naturally bear approximately two thirds of their weight on their forelimbs; thus, representing the brunt of musculoskeletal development and engagement during normal physical activity (3, 4). The uneven distribution of weight along with common hip and spinal anomalies may impact muscle development and maintenance of the hindlimbs and core, predisposing these areas to injury or degenerative changes during moderate and vigorous activities encountered in the majority of working dog careers (5–7). Development of a dog's musculoskeletal system in the optimal biomechanical alignment through whole body proprioception, muscle stability and strength of the core, forelimbs, and hindlimbs, and mobility may decrease a dog's susceptibility to, the severity of, or the recovery time for an injury.

**Table 1:** Definitions of foundational canine fitness components.

**Foundational fitness definitions**	
**Term**	**Definition**
Foundational canine fitness	The mobility, muscle stability (core, forelimb, hindlimb), muscle strength, and proprioception required to perform basic physical activities such as running, jumping (up and down), navigating unstable surfaces, and quickly or abruptly changing direction that a dog needs to perform its job-related tasks.
Mobility	The ability to move the body without limitation from joints, muscles, tendons, or other connective tissues.
Strength	The ability of a muscle group to exert maximal force.
Stability	The ability for the muscles and tissues supporting a joint to resist unwanted or abnormal movement.
Forelimb stability	The ability to maintain the shoulder, elbow, and carpus in biomechanically optimal positions.
Hindlimb stability	The ability to maintain the hip, stifle, and tarsus in biomechanically optimal positions.
Core stability	The ability to maintain the spine (cervical, thoracic, lumbosacral) in biomechanically optimal positions.
Proprioception	The perception or awareness of the position and movement of the body (12).

This canine foundational fitness program is designed to be incorporated into an established working dog training program (e.g., search and rescue, law enforcement, military, etc.). The dog's career training develops the baseline cardiovascular endurance and skill related components of career-specific fitness. However, additional career-specific fitness training should be pursued to optimize career performance. A canine foundational fitness program for working dogs is designed to incorporate exercises that require minimal additional training of the dog and handler, include clear incremental exercise progressions, utilize easily accessible and low-cost equipment, and should also be applicable to a wide variety of working dog careers.

### Foundational Fitness Assessment

One of the biggest challenges in evaluating the current level of canine fitness and the impact of any intervention, such as a fitness program, is the establishment of a formalized, repeatable method for assessment. To date, there are no peer-reviewed, published studies of systematic foundational programs with assessments specifically for working dogs. The methods for creating a foundational fitness assessment should produce scientifically valid, reliable, and reproducible canine fitness data (8–10). Clear and objective outcome variables, such as duration of exercise, number of repetitions, or distance traveled are critical. The assessment should be designed to account for intra-dog and inter-dog variability and learning effect of the dog, but lead to results that allow the dog to be compared to the population (9). The individual performing the assessment should not require extensive experience, thereby maximizing the intra-rater or inter-rater reliability. Minimizing human interaction with the dog during the assessment is important to prevent skewing the data. Finally, skills being assessed should directly translate to the realistic and operational athletic requirements of working dogs (8, 11).

### The Fit to Work Program

The Fit to Work (FTW) foundational exercises and associated assessment method presented here are designed to create a formalized, highly reproducible, and inexpensive method to create the first stage of a balanced fitness program for working dogs. The goals of the program are to meet the foundational fitness requirements of working dogs that are not currently addressed in the traditional training disciplines. The program was designed with the realistic expectations and time constraints of the working dog training and utilization worlds. To address the gap in knowledge and lack of additional training time, the FTW program contains a discrete number of exercises, standardized progression steps, and defined criteria for progression. The FTW program significantly reduces the requirement for learned behaviors to shorten the learning curve and enable a novice working dog handler to develop their dog's physical fitness. We will present each of the components of the program, their description, the purpose of the exercise, the known or anticipated role in performance and injury prevention, a recommended progression of difficulty, a brief summary of contraindications, and suggested training approaches.

## Methods

### Pilot Implementation

All dogs included in the implementation below are owned by the Penn Vet Working Dog Center, School of Veterinary Medicine, University of Pennsylvania. The Fit to Work (FTW) program is included in the Penn Vet Working Dog Center (PVWDC) Puppy Foundation Program protocol 804547 approved by The University of Pennsylvania Institutional Care and Use Committee.

The FTW program was piloted on a closed population of 31 dogs in training for careers in search and rescue, law enforcement, single-purpose detection, and medical detection. Each dog was fostered on nights and weekends by an individual or family who did not perform any FTW activities. The dogs ranged in age from 2 months to 6 years of age and were of the Labrador Retriever (*n* = 15), German Shepherd Dog (*n* = 8), Belgian Malinois (*n* = 3), Dutch Shepherd (*n* = 3), Small Munsterlander (*n* = 1), and Doberman Pinscher (*n* = 1) breeds. The dogs were assigned to 5 full-time trainers who each had responsibility for 3–8 dogs. The training team was assisted by ~20 part-time undergraduate interns and ~30 adult volunteers.

### Foundational Fitness Training Structure

The foundational fitness training consists of a series of daily and three times weekly exercises. Daily exercises develop posture via the Posture Down and Posture Sit and enhance mobility via the Warm-Up and Cool-down exercises prior to and immediately after any moderate or vigorous activities. Three times a week, the dog participates in the foundational fitness exercises which focus on strength, stability, balance, and proprioception. These exercises are divided into two circuits consisting of three exercises per circuit. Circuit One consists of the Posture Down or Chipmunk, the Squat, and the Back-up. Circuit Two consists of the Plank, the Pivot, and the Back-up. Each circuit is performed twice either consecutively or in an alternating fashion. Each exercise within the circuit is performed for 30–60 s. The entire training session takes between 15 and 20 min excluding equipment setup and break down time.

A summary of the foundational fitness exercises, their fitness modality targets, and recommended reward methods can be found in Table 2.

**Table 2:** Summary of the foundational fitness exercises and their primary and secondary targets and the preferred method of rewarding during the exercise.

**Foundational fitness exercise summary**												
	**Exercise name**	**Primary and secondary targets**								**Reward method**		
		**Mobility**	**Core stability**	**Core strength**	**Hindlimb stability**	**Hindlimb extension**	**Forelimb stability**	**Forelimb extension**	**Proprioception**	**T**	**IF**	**CF**
Daily exercise	Figure-8	P	S		S		S		S	X	X	
	Paws-up	P	S		S				S	X	X	
	Four-position cookie stretch	P							S	X		X
	Posture sit	S	P	S	S				S	X		X
Weekly circuit	Posture down	S	P	S	S				S			X
	Chipmunk		P	P	S		S		P			X
	Plank		P	S	S		S	S	S			X
	Pivot		P	S^*^	P		S		S	X	X	
	Squat		S	S^*^	S	P			S	X	X	
	Back-up		S		S		S	P^*^		X	X	

*Primary and secondary targets key: P, primary target; S, secondary target*;

* *at the higher progression levels. Reward method key: T, Toy; IF, intermittent food reward; CF, continuous food reward*.

### Foundational Fitness Exercises

#### Warm-Up and Cool-Down

##### Description

**Figure 1:**
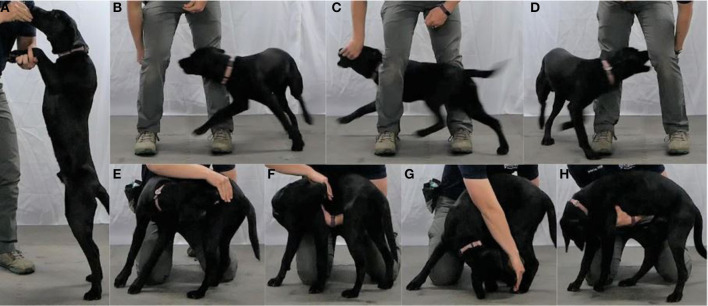
**(A)** Shows the Paws-up. **(B–D)** Show the Figure-8. **(E–H)** Show the Four-Position Cookie Stretch [**(E)** Nose to hip, **(F)** Nose to shoulder, **(G)** Nose to rear foot, and **(H)** Nose to chest].

To complete the Warm-up, the dog walks for 30 s, trots for 30 s, performs a Paws-up for 15 s on the handler's arm or an object (Figure 1A), and performs 3 Figure-8s (Figures 1B–D) between the handler's legs or around two objects or people set 45–90 cm (18–36 in) apart. The Warm-up should take ~90 s to complete. To complete the Cool-down, the dog walks for 30 s while the handler observes for any physical or behavioral abnormalities, performs a Paws-up for 15 s, and performs a Four-Position Cookie Stretch (Figures 1E–H) on each side. Finally, the handler checks the dog's paws, pads, and nails for signs of injury. The Cool-down should take ~120 s to complete.

##### Purpose

The Warm-up should be completed before all activities to prepare the dog's body for the upcoming movements. The walk and trot portion of the Warm-up progressively increase body temperature and tissue mobility (12). The Paws-up targets the hip and abdominal tissues (predominantly the iliopsoas, psoas major, and rectus abdominis muscles) for extension, and the Figure-8 prepares the neck and trunk tissues (predominantly the lateral muscles of the neck, extrinsic muscles of the forelimb, internal and external abdominal oblique muscles, and epaxial spinal muscles) for lateral movement.

The Cool-down should be performed after all activity to maintain and increase mobility while tissues are warm and to identify any injuries sustained during training. The walk portion of the Cool-down allows the dog's heart rate and breathing to begin to decrease, and gives the handler the opportunity to identify any lameness. The Paws-up targets the mobility of hip and abdominal tissues, and the Four-Position Cookie Stretch targets the mobility of the neck and trunk. Finally, checking the dog's paws, pads, and nails after training allows rapid identification of issues in these injury-prone areas.

##### Role in performance and injury prevention

The walk and trot portions of the Warm-up increase blood flow and oxygen delivery to muscles and connective tissues and increase body temperature to prepare for higher-intensity activity (13, 14). The Paws-up and Figure-8 may optimize movement and decrease injury by allowing the dog access to an increased tissue range of motion (15–17). Both movements may also increase neuromuscular activation and thus athletic performance (18, 19). The entire Cool-down gives the dog's body time to recover from activity before returning to rest and the handler an opportunity to identify injuries that were not evident when the dog was engaged in activity. While post-exercise stretching appears to have limited effects on muscle soreness (20), the increased tissue mobility from the Paws-up and Four-Position Cookie Stretch may decrease future tissue injury risk (21, 22) and increase future performance (23). Checking the dog's paws, pads, and nails allows early identification and rapid treatment of performance-limiting injuries.

##### Contraindications

Without guidance from a veterinarian, active exercise and thus the Warm-up and Cool-down are not recommended for dogs with suspected musculoskeletal abnormalities or cardiorespiratory disease. With supervised rehabilitation, the use of and adaptations to the Warm-up and Cool-down can be customized to the dog's injury or condition.

##### Progression

The range and thus the intensity of the Paws-up may be increased by adjusting the height of the handler's arm or the object used. The intensity of the Figure-8 may be increased by decreasing the width between the handler's legs (or between the objects that the dog is navigating) or by increasing the speed of execution. The range and thus the intensity of the Four-Position Cookie Stretch may be increased by extending the dog's nose closer to each targeted position or by increasing the duration at each position.

##### Training

While many ways exist to perform or train the Warm-up and Cool-down exercises, we have found the following to work well for our population of working dogs. To train the Paws-up on an arm, have the dog sit, kneel next to them, scoop your forearm under their forelimbs, and use a toy or food reward to lure them into a stand as you slowly rise. Use the toy or food reward to maintain them in the Paws-up position for the desired duration. Common technique errors include rewarding too high above the dog's head which overextends the dog's neck and allowing the dog to rest a significant portion of their weight on your forearm.

To train the Figure-8, start with your feet approximately twice your shoulder width distance apart. Use a “Touch” command or lure the dog between your legs, then with alternating hands direct the dog in one direction (e.g., clockwise around your right leg), then back through your legs and around the other leg in the opposite direction (e.g., counterclockwise around your left leg). Common technique errors include inadequate rate of reinforcement, giving improperly timed commands and imprecise luring, all of which prevent the dog from sharply turning around your legs. Good form also requires good handler form, standing up straight is the goal.

To train the Four-Position Cookie Stretch, lure the dog in a standing position so that its spine is aligned perpendicular to your spine. Gently place your opposite hand under their thorax or abdomen to maintain their position against you. Lure their nose away from your body, in a plane parallel to their spine to their hip, mark, and reward. Then lure their nose to their hip and then gradually move it as close to their shoulder as possible, while maintaining it in the plane parallel to their spine, mark, and reward. Lure their nose to their hip and then move the lure distally until their nose is as close to their rear foot as possible, mark, and reward. Finally, lure their nose between their forelimbs and as close to their chest as possible, mark, and reward. A common technique error is not maintaining the dog's position against your body which allows them to decrease the intensity of the exercise.

#### Posture Sit and Posture Down

##### Description

**Figure 2:**
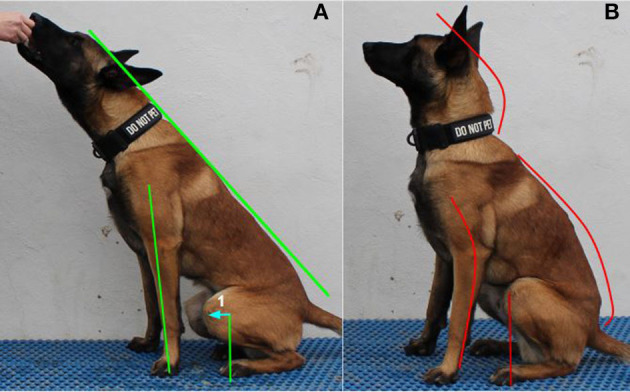
**(A)** Shows the Posture Sit in the correct position. Note the straight line from the head to the base of the tail. The forelimb is extended in a straight line. The stifle is dorsal to or just cranial to the digits (line 1 shows the range of the stifle over the digits). **(B)** Shows the Posture Sit in the incorrect position. Note the rounded forelimb and spine (cervical, thoracic, and lumbosacral). The stifle is positioned caudal to the digits.

To perform the Posture Sit, the dog should sit with its coxofemoral joint, stifle, tarsus, and hindlimb digits in the same straight sagittal plane (Figure 2). The forelimbs should be half a stride length in front of the hindlimb digits. The forelimb paws should be directly under the shoulder and should remain on the ground throughout the exercise. The shoulder (glenohumeral joint), elbow, carpus, and forelimb digits should be aligned in the same straight sagittal plane as the hindlimbs. To achieve the Posture Sit position, the dog extends its spine and rolls its pelvis forward to form a straight line from the nose to the base of the tail (Figure 2A). During the movement, the dog's stifles should move dorsal to or just cranial to the hindlimb digits.

**Figure 3:**
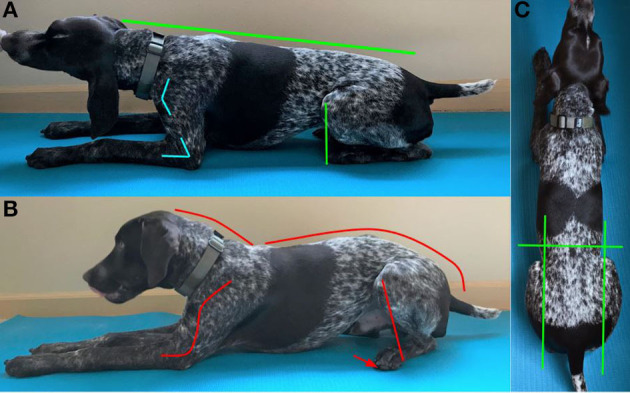
**(A)** Shows the Posture Down in the correct position. Note the straight line from the head to the base of the tail. The forelimb is flexed at the elbow and shoulder. The stifle is dorsal to or just cranial to the digits. **(B)** Shows the Posture Down in the incorrect position. Note the extended elbow and rounded spine (cervical, thoracic, and lumbosacral). The hindlimbs are abducted and the stifle is caudal to the digits. The red arrow indicates the abduction of the hind foot. **(C)** Shows the correct position of the Posture Down from above (the dorsal view) the dog. All four ipsilateral limbs are aligned in a sagittal plane, and the hindlimb digits are obscured by the stifles.

To perform the Posture Down, the dog should lay down so that the ipsilateral right and left limbs are within the same sagittal plane, often referred to as a “sphinx” position (Figure 3). To achieve the Posture Down position, the dog flexes its shoulder and elbow 10–20° depending on size of the dog, rolls its pelvis forward, and extends its spine to form a straight line from the nose to the base of the tail (Figure 3A). During the movement, the dog's stifles should move dorsal to or just cranial to the hindlimb digits.

##### Purpose

Performing the Posture Sit and Posture Down primarily targets stabilizer muscles of the spine (sagittal extension) and abdominal engagement (sagittal flexion). The primary muscle groups of the Posture Sit and Posture Down are the cervical spine extensor muscle (splenius), trunk extensor muscles—epaxials (transversospinalis, longissimus, iliocostalis), latissimus dorsi, hip stabilizers (gluteal, psoas major, iliacus, adductor, and piriformis muscles), and abdominal muscle (rectus abdominis). Proprioception of spine, shoulder, elbow, lumbosacral, coxofemoral joint, and stifle are secondarily developed during both the Posture Sit and Posture Down.

##### Role in performance

Performing the Posture Sit and Posture Down strengthens the dog's core stability musculature and supports optimal biomechanical alignment by creating a proprioceptive memory of correct posture (5, 24, 25). Promoting correct posture in a sit and down position establishes the foundation necessary to perform activities safely (25). All dogs benefit from correct posture whether in athletic performance, tactical operations, or as a household pet.

##### Role in injury prevention

Engaging in Posture Sits and Posture Downs may prevent repetitive stress on the spine resulting in kyphosis and susceptibility to traumatic or alignment-related injuries (22). Additionally, observation of a dog's ability to achieve the proper Posture Sit and Posture Down can act as a screen for subtle injuries. A dog that is reluctant to extend its spine in the Posture Sit or Posture Down may be experiencing lower back pain. A dog that is reluctant to sit or down in a straight sagittal plane and abducts a limb may be experiencing hip or stifle pain. Recognition of these subtle changes can lead to earlier diagnosis of an injury and prevention of further injury.

##### Progression

Teaching correct posture development in both the sit and down positions when the dog is a puppy (8 weeks) and maintained throughout the dog's life is highly encouraged. For healthy dogs, progression in the Posture Sit and Posture Down is achieved by increasing the duration of holding the correct position and then destabilizing the surface on which the dog is performing the exercise. For beginner dogs, the duration may be as short as 1–2 s. The dog is expected to maintain a 30 s hold in the correct position before progressing to an unstable surface. An unstable surface should be a flat platform with the destabilization component located underneath the platform. Performing this exercise on a flat surface allows the trainer to evaluate if the dog is not remaining in a proper position and/or is consistently favoring one side over another. If the exercise is performed on two unstable surfaces such as balance discs alone, the dog can make subtle changes in body posture that may result in asymmetric muscle development and lack of engagement of the smaller secondary musculature.

##### Contraindications

The Posture Sit and Posture Down are safe for healthy dogs of all ages. Caution in progression of the Posture Sit and Posture Down should be taken with suspected spine, hip, or stifle abnormalities. While critical to the rehabilitation process of many musculoskeletal injuries, the Posture Sit and Posture Down should only be performed for these patients under the guidance of the dog's veterinarian.

##### Training

To train the Posture Sit and Posture Down, have the dog perform a correct (square) sit or (sphinx) down position as described above. Placing the dog on a narrow platform or between two objects will encourage the dog to align all limbs in the sagittal plane. Once in the proper square sit or sphinx down, a treat or toy lure at a dog's resting nose level height should be utilized to encourage the dog to move forward slightly [2–7 cm (1–3 in) depending on the dog's size]. The dog should be lured until the spine is straight and the stifles are dorsal to or just cranial to the hindlimb digits. If the treat or toy lure placement is too high, the dog will attempt to stand. If the treat or toy lure placement is too low in the Posture Down, the dog will lift its hocks or attempt to crawl. If the treat or toy lure placement is too low in the Posture Sit, the dog will attempt to lay down or will round its back. Time the delivery of the reward to the dog based on the dog's progression level and deliver the reward while the dog is holding the correct posture position.

#### Pivot

##### Description

**Figure 4:**
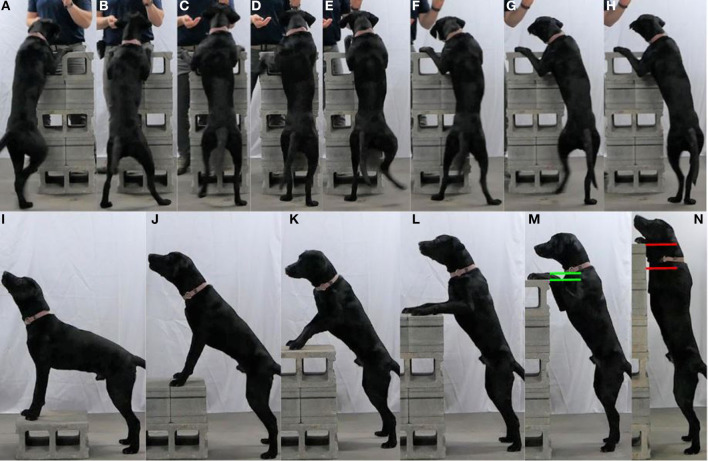
**(A–H)** Illustrate the Pivot. **(I–N)** Show progression of the Pivot [**(I)** Level 1, **(J)** Level 2, **(K)** Level 3, **(L)** Level 4, **(M)** Level 5 (maximum for this dog), and **(N)** Level 6 (too high for this dog)].

To perform the Pivot, the dog places its forepaws on an elevated stable object (e.g., standard concrete block) and steps laterally (sidesteps) with its hindpaws around the object both clockwise and counterclockwise (Figures 4A–H). The Pivot is performed continuously in one direction for a specific duration or number of rotations and then repeated in the opposite direction after a rest interval.

##### Purpose

The Pivot primarily develops the dog's hip, stifle, and tarsus stability. Core stability (predominantly sagittal and lateral flexion), hindlimb proprioception, and hip, stifle, and tarsus extension are secondarily developed. Hip stability is provided by the hip abductors (superficial, middle, and deep gluteal muscles) and adductors (adductor, gracilis, semimembranosus, sartorius, and pectineus muscles). Stifle and tarsus stability may also benefit from the improved control over the lower extremity provided by the stable hip during movement (26).

##### Role in performance

The Pivot strengthens the dog's hip stability musculature and supports optimal biomechanical alignment of the hip, stifle, and tarsus when the dog is moving and stationary (26). A stable hip may increase power generation when jumping or sprinting (27), provide effective lateral force and stable footing when turning (28), and align the hips for subsequent movements when landing from a jump (29). A dog that is required to perform movements with the forelimbs elevated, weight shifted to the hindlimbs, and the hip extended (e.g., searching elevated surfaces or vehicles) may also benefit from the Pivot.

##### Role in injury prevention

Stable hips developed by the Pivot may prevent traumatic (30) or alignment-related injuries and assist in limiting the progression of orthopedic disease (31). A dog that missteps or begins to slip into a splayed hindlimb position may incur an iliopsoas muscle, hip adductor group, or hip joint injury. If this dog has more stable hips from training the Pivot, it may have a better chance of recovering its footing and preventing injury (30). Increased hip stability helps maintain optimal motion of the hip and stifle joints and may reduce injuries caused by poor joint alignment (32, 33). Although there are no clinical trials to evaluate the impact, dogs with early dysplastic hips may benefit from increased muscular support and reduced joint instability, and dogs with stable cruciate ligament disease may benefit from the improved lower extremity control.

##### Progression

For mature dogs, the Pivot is usually trained on a 20 cm (8 in) high and 40 cm (16 in) square object (e.g., two standard concrete blocks) (Figure 4I), although puppies under 3–4 months of age or small breed dogs may benefit from an object that is only 20 cm (8 in) square (e.g., two standard bricks). Once the dog is able to complete three revolutions in 30 s in each direction, the object height is increased by 20 cm (8 in) (Figures 4J–M). Elevation of the object height progressively shifts a greater percentage of the dog's weight onto their hindlimbs. This process is continued until the next height progression would result in the dog's forepaws being elevated higher than the shoulder joint (Figure 4N). Further progression is primarily provided by external weight in the form of a weight vest with weight increments scaled to the weight of the dog (e.g., for a 20 kg (44 lb) dog start with 2.3 kg (5 lb) of external weight). Alternate methods of progression include destabilizing the hindpaw surface or increasing hindlimb lateral resistance (water or exercise band).

##### Contraindications

Without guidance from a veterinarian, the Pivot is not recommended for dogs with suspected spine, hip, stifle, tarsus, or hindpaw abnormalities. With supervised rehabilitation, the Pivot can be adapted to the dog's injury or condition.

##### Training

While there are many ways to perform or train the Pivot, the following works well for our population of working dogs. First, have the dog place their forepaws on the elevated object, stand next to the dog's flank, provide the verbal cue to “Step,” provide gentle body pressure by moving into the dog, and then mark and reward any lateral movement of the hind foot while the forelimbs remain on the object. Hold the reward in the right hand when performing the Pivot clockwise and in the left hand when performing the Pivot counterclockwise. Work toward having the dog moving at least a quarter rotation ahead of you and rewarding after each quarter rotation. A well-trained dog should perform the Pivot in a position 180° from the handler and be able to complete a 30 s interval prior to reward. Common technique errors include relying solely on body pressure after an initial learning period, using the hand closest to the dog to reward, rewarding too far above or away from the dog's mouth or too infrequently, or allowing the dog to move too quickly and skip with or cross their hind feet. A soft high-value reward (e.g., small piece of cheese) assists our population of working dogs to rapidly swallow the reward and resume movement.

#### Plank

##### Description

**Figure 5:**
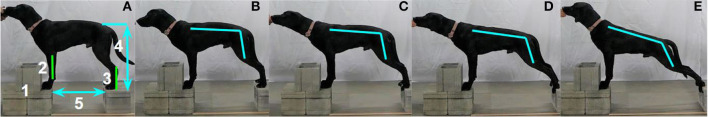
**(A)** Shows the Plank setup (1 shows the recommended three-block front object), starting position [2 illustrates a vertical distal forelimb and 3 illustrates vertical metatarsals (rear pastern)] and measurements (4 is the hip height and 5 is the distance between the objects). **(B–E)** Show progression of the Plank [**(B)** Level 2, **(C)** Level 4, **(D)** Level 6, and **(E)** Level 8].

To perform the Plank, the dog stands with its forepaws on one stable object (e.g., standard concrete block) and its hindpaws on a second stable object of equal height. Place (or cue the dog to place) their forepaws on the rear edge of the front object and their hindpaws on the front edge of the rear object. Adjust the distance between the objects until both the dog's distal forelimbs (elbow to carpus) and metatarsals (rear pastern) are vertical (Figure 5A, Lines 2 and 3). Measure and record the distance between the objects (Figure 5A, Line 5). Next, measure the dog's height from the top of the object to the dorsum of the dog directly above the coxofemoral joint (Figure 5A, Line 4) and use this to determine the dog's height-adjusted movement increment (Table 3). Then, move the two objects apart to the desired level, and the dog holds the position for the specified duration or until they step down. A proper Plank is performed when the distal forelimbs are maintained in a vertical position and the hindpaws are in contact with the rear object (Figures 5B–E).

**Table 3:** Plank hip height measurements, corresponding Plank level increment, total distance for each hip height and Plank level, and approximate hip angle achieved.

**Plank hip height, increment, and level All measurements are in centimeters**										
**Hip Height**	**Increment**	**Level 1**	**Level 2**	**Level 3**	**Level 4**	**Level 5**	**Level 6**	**Level 7**	**Level 8**	**Level 9**
Approx. hip angle		93°	96°	99°	103°	107°	112°	122°	135°	150°
<20	1	1	2	3	4	5	6	7	8	9
20–26	1.5	1.5	3	4.5	6	7.5	9	10.5	12	13.5
27–33	2	2	4	6	8	10	12	14	16	18
34–40	2.5	2.5	5	7.5	10	12.5	15	17.5	20	22.5
41–47	3	3	6	9	12	15	18	21	24	27
48–54	3.5	3.5	7	10.5	14	17.5	21	24.5	28	31.5
55–61	4	4	8	12	16	20	24	28	32	36
62–69	4.5	4.5	9	13.5	18	22.5	27	31.5	36	40.5
>70	5	5	10	15	20	25	30	35	40	45

##### Purpose

The Plank primarily develops a dog's core stability (predominantly resistance to thoracic and lumbar spine extension). Isometric elbow and carpus extension are secondarily developed. Core stability in the Plank is predominantly provided by the rectus abdominis, external abdominal oblique, iliopsoas, and psoas minor muscles. In humans, the spinal extension muscles have also been shown to play a role (34, 35).

##### Role in performance

The Plank strengthens the muscles that provide stability to a dog's spine. This increased stability may enhance the dog's ability to generate whole-body power (36–38), run (39–42), and perform single-leg movements (43). While improved core stability has differing effects on agility in humans (44, 45), the differences in anatomy may enhance the role of core stability for canine agility. A dog performing movements with the forepaws and hindpaws on separate surfaces, the forepaws on elevated surfaces, or any paw on unstable surfaces may benefit from training the Plank (34, 46).

##### Role in injury prevention

Training movements that develop a dog's spinal stability may increase the likelihood of maintaining optimal biomechanical alignment when gravity or the motion of the dog's body cause thoracic or lumbar spine hyperextension. A dog that repeatedly hurdles obstacles (e.g., agility or law enforcement), spends prolonged time in a forepaw-elevated position (e.g., searching vehicles or elevated surfaces), traverses unstable surfaces that predispose it to a fall, (e.g., disaster search and rescue) encounters powerful force to the spine (e.g., criminal apprehension), or is at risk of spine injury or intervertebral disc disease may benefit from training the Plank.

##### Progression

The Plank is usually trained in intervals of up to 30 s at a particular level. The dog is given 45 s in which to accumulate 30 s of proper Plank. The 30 s duration time is paused when the dog moves out of the proper position (by shifting backwards so the distal forelimbs are no longer vertical or moving a hindpaw off the rear object) and the timer is resumed when the proper position is achieved again. The interval is stopped when the dog accumulates 30 s, the 45 s time elapses, or the dog steps off either object.

Once a dog is able to accumulate 30 s of proper Plank within the 45 s window, the objects can be moved to the next level (Figures 5B–E). This process is continued until the dog completes 30 s at Plank—Level 9. Further progression is primarily provided by external weight in the form of a weight vest with weight increments scaled to the weight of the dog [e.g., for a 20 kg (44 lb) dog start with 1.1 kg (2.5 lb) of external weight]. Alternate methods of progression include destabilizing either the forepaw surface, the hindpaw surface, or both.

##### Contraindications

Without guidance from a veterinarian, the Plank is not recommended for dogs with suspected spine or hip abnormalities. With supervised rehabilitation, the Plank can be adapted to the dog's injury or condition.

##### Training

While there are many ways to perform or train the Plank, the following works well for our population of working dogs. Position the stable objects (e.g., standard concrete blocks) at the starting position (Level 0), and have the dog step onto the rear object and walk across to the front object. For some dogs, placing an additional object between the front and back objects will facilitate the dog walking across the gap. An extra object may be used on top of the front object to assist the dog with maintaining its forepaws on the inside edge (Figure 5A). Gently slide the rear object to the desired level (an assistant may be useful for this step). Use a “Touch” command or a food reward to obtain and maintain the proper Plank position. Common technique errors include failure to adjust the dog's feet to the inside edges of the objects and improper positioning of the reward so the dog's neck is improperly aligned or the forelimbs are not vertical. A continuous high-value reward (e.g., frozen peanut butter in a cup) assists our population of working dogs in maintaining the proper position.

#### Chipmunk

##### Description

**Figure 6:**
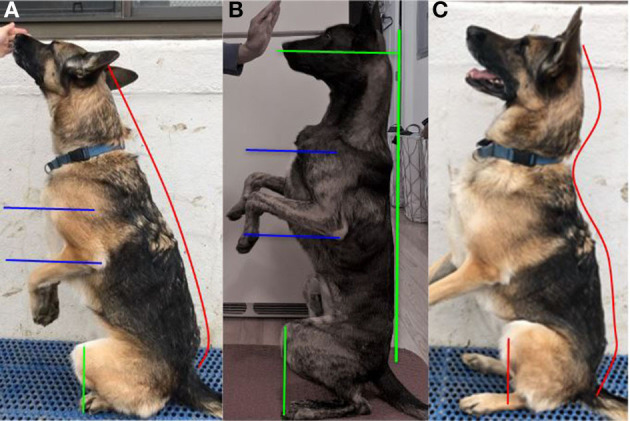
**(A)** Shows the beginning progression of the Chipmunk—note the stifle dorsal to the digits. This position is held for <2 s. **(B)** Shows the correct Chipmunk position. The spine is straight, the stifles are dorsal to the digits, the carpi are suspended between the shoulders and elbows, and the head is in a neutral position. **(C)** Shows an incorrect Chipmunk. The spine is rounded in all three segments, the stifle is caudal to the digits, the dog's entire weight is resting on the ischium of the pelvis that is touching the ground.

The Chipmunk is also known as the “beg,” “sit pretty,” “sit up,” or “sit erect.” To perform the final correct position, the dog must have a square sit as described in the Posture Sit exercise. Once in a sit, the dog's forelimbs are suspended off the ground with the carpi suspended between elbow and shoulder height, the dog maintains a straight spine, and holds their head in a neutral position facing forward (Figure 6B).

##### Purpose

The Chipmunk primarily develops the dog's core stability (resistance to spinal extension, sagittal flexion, and transverse flexion). Secondarily, isometric shoulder and elbow contraction and minor concentric contraction of quadriceps muscles are involved. The primary stability muscles engaged during the Chipmunk are the rectus abdominis, external abdominal oblique, iliopsoas, transversospinalis, longissimus, and iliocostalis muscles.

##### Role in performance

The Chipmunk strengthens the muscles that provide stability to a dog's spine (4). Along with muscle development, the Chipmunk enhances the dog's whole body balance and proprioception (4). This increased stability, balance, and proprioception may enhance the dog's ability to produce power, improve endurance, and enhance agility (46, 47).

##### Role in injury prevention

Training exercises that enhance a dog's balance, proprioception, and core strength may protect them during uncontrolled movements which result in body misalignment, spinal hyperextension or compression, such as jumping, apprehension training, or ladder climbing (41, 43).

##### Progression

A Chipmunk should only be attempted once the dog has achieved consistent proper Posture Sits and Posture Downs. Achievement of the correct Posture Sit and Posture Down ensures that the dog has sufficient body awareness and muscle development of the epaxial and abdominal muscles. As described in the training section below, the dog should gradually be taught to engage its core in short training sessions before expecting the final Chipmunk position. Once a dog can achieve the proper form of a Chipmunk for a duration of 30 s (Figure 6B), it can progress to more advanced levels. To increase the difficulty of a Chipmunk, move the lure left and right, encouraging the dog to shift its weight. The further away from the dog that the lure is located, the more difficult the exercise becomes. Once the dog can maintain its balance for 30 s while weight shifting, they can progress. The final progression is to perform the Chipmunk on an uneven or unstable surface. Progression of the Chipmunk requires patience, hypervigilance of posture, and commitment to long-term training.

##### Contraindications

The Chipmunk is not recommended for dogs with suspected spine (cervical, thoracic, or lumbosacral), hip, or stifle abnormalities, unless under direct supervision of a veterinarian.

##### Training

Training sessions for the Chipmunk should be short, and the dog should not perform more than 3–6 repetitions during any single session. To begin to train the Chipmunk, place the dog in a Posture Sit and place a lure just above the dog's head to encourage lifting of the forelimbs. Reward as soon as the forelimbs leave the ground [<15 cm (6 in)] (Figure 6A). Once the dog lifts its forelimbs to 15 cm (6 in), the next progression is to have the dog lift the forelimbs to a height between the elbow and shoulder. Next, encourage the dog to progress to holding themselves with a straight spine, square sit, neutral head position, and stifles dorsal to the hindlimb digits. If the dog's stifles are not dorsal to the hindlimb digits the dog is not engaging its core musculature appropriately. Do not increase the duration of the Chipmunk hold until the dog can consistently hold itself in the proper posture (Figure 6B). If proper posture is achieved, then progress to the more advanced Chipmunk described in the progression section. A dog may require weeks to months to attain the proper posture in the Chipmunk. Signs that a dog is fatiguing are muscle fasciculations, reluctance to lift forelimbs, attempts to place forelimbs on an object/handler, or the stifles consistently moving caudally away from the hindlimb digits.

#### Squat

##### Description

**Figure 7:**
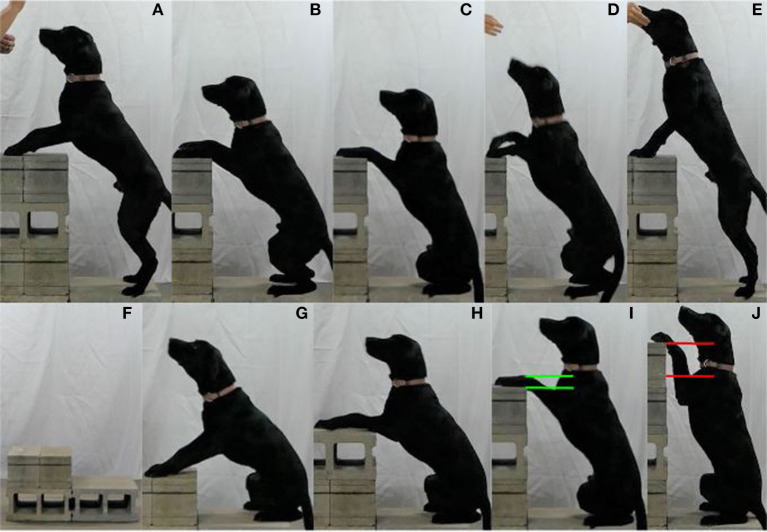
**(A–E)** Illustrate the Squat. **(F)** Shows the restricted area platform. **(G–J)** Show progression of the Squat [**(G)** Level 1, **(H)** Level 2, **(I)** Level 3 (maximum for this dog), and **(J)** Level 4 (too high for this dog)].

To perform the Squat, the dog places its forepaws on an elevated object, sits onto a restricted area platform, and then returns to the starting position (Figures 7A–E). The Squat is performed for a specific duration or number of repetitions.

##### Purpose

The Squat primarily develops the dog's hip, stifle, and tarsus extension strength (48). Core stability (predominantly sagittal flexion) and hindlimb proprioception are secondarily developed. Hip extension is primarily provided by the gluteal (superficial, middle, and deep), semitendinosus, and semimembranosus muscles. The gracilis, piriformis, and quadratus femoris muscles also contribute to hip extension. Stifle extension is primarily provided by the quadriceps femoris, sartorius, tensor fasciae latae, and biceps femoris muscles. Tarsal extension is primarily provided by the gracilis, biceps femoris, and semitendinosus muscles.

##### Role in performance

Forceful hip, stifle, and tarsus extension developed from performing the Squat may increase the dog's ability to jump, sprint, and change direction. Dogs needing to jump up, onto an elevated surface, over an object, or across a gap may benefit from the Squat. Dogs rising from a down or sitting position or those rapidly accelerating while sprinting can benefit from the Squat. Dogs doing single-leg movements, climbing stairs, or rapidly changing direction may also perform better as a result of incorporating the Squat into their fitness plan (49).

##### Role in injury prevention

Strong hips, stifles, and tarsi developed by the Squat may be less prone to injury (50). Increased hip musculature may reduce the risk of hip dislocation and provide stability to a dysplastic hip (31). Developing stifle extension musculature may reduce cranial movement of the tibia relative to the femur and provide increased support to the cranial cruciate ligament. Dogs may also experience fewer or lower severity tarsal sprains or common calcanean tendon injuries (51).

##### Progression

For mature dogs, the Squat is usually trained on a 20 cm (8 in) high stable object (e.g., standard concrete block) (Figure 7F), although puppies under 3–4 months of age or small breed dogs may benefit from an object that is only 10 cm (4 in) high (e.g., standard brick). Once the dog is able to complete 7 repetitions revolutions in 30 s, the object height is increased by 20 cm (8 in) or by 10 cm (4 in) for young or small breed dogs (Figures 7G–I). Elevation of the object height progressively shifts a greater percentage of the dog's weight onto their hindlimbs. This process is continued until the next height progression would result in the dog's forepaws being elevated higher than the shoulder joint at the bottom of the movement (Figure 7J). Further progression is primarily provided by external weight in the form of a weight vest with weight increments scaled to the weight of the dog [e.g., for a 20 kg (44 lb) dog start with 2.3 kg (5 lb) of external weight]. Alternate methods of progression include destabilizing the hindpaw surface.

##### Contraindications

Without guidance from a veterinarian, the Squat is not recommended for dogs with suspected spine, hip, stifle, or tarsus abnormalities. With supervised rehabilitation, the Squat may be adapted to the dog's injury or condition.

##### Training

A restricted area platform typically 40 cm (16 in) square (e.g., two standard concrete blocks) is used to maintain the forepaws on the elevated object and the hindpaws at a consistent distance from the object. While there are many ways to perform or train the Squat, the following works well for our population of working dogs. Have the dog step onto the platform and then onto the elevated object. Provide the verbal cue to “Sit” and use a food reward or toy to guide the dog's nose backward. Mark and reward any flexion of the hips, stifles, or tarsi. Progress toward a full sit with both forepaws on the elevated object and the majority of the hindlimb below the hock on the platform. Use a “Touch” command or lure the dog to return to the starting position. A soft high-value reward (e.g., small piece of cheese) assists our population of working dogs to rapidly return to the starting position, swallow the reward, and begin the next repetition.

#### Back-up

##### Description

**Figure 8:**
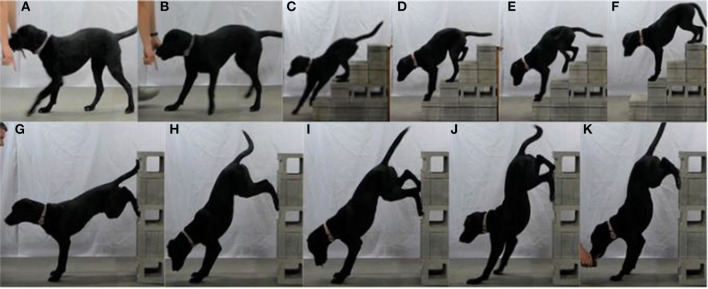
**(A,B)** Show the first progression of Back-up on the ground. **(C–F)** Shows a more advanced progression of the Back-up, backing up stairs. **(G–K)** Shows another advanced progression of the Back-up, backing up a vertical wall.

To perform the Back-up exercise, beginning at a walk, the dog moves in a backwards or reverse motion by picking up and pushing off the ground with each foot to propel themselves backwards. The dog's spine should remain parallel with the floor during the entire movement (Figure 8A).

##### Purpose

Back-up is utilized for proprioception of the hindlimbs and primary targeting of the biceps brachii and quadriceps muscles (4, 24). The dog's normal gait is a pulling mechanism that primarily engages the triceps and hamstrings. The swing phase of the gait, which primarily involves the biceps and quadriceps muscles, is a passive motion (23, 45, 47). Backwards walking transitions the active and passive phases of the walking and trotting gait to build the biceps brachii, brachialis, and hamstring (semimembranosus and semitendinosus) muscles (24).

##### Role in performance

Performing the Back-up exercise focuses on the extensors of the hindlimb while complementing the flexion focused Squat exercise (24, 49, 52). The enhanced proprioceptive and activation of neuromuscular pathways that are not naturally targeted in a dog may increase athletic performance. Placement of backwards walking between the flexion focused exercises of the fitness circuit allows for active recovery time for the flexor muscles. The whole-body training approach may lead to balanced power, stamina, and enhance proprioception (24, 49, 52).

##### Role in injury prevention

Training of secondary muscle groups, activation of secondary gait neuromuscular pathways, and enhancing hindlimb proprioception may protect a dog from injury (3, 22, 28, 48, 49). Utilizing the Back-up during the fitness circuit maintains warmer tissue temperatures which improves tissue elasticity decreasing the risk of tissue damage during the remainder of the exercises (19, 20).

##### Contraindications

The Back-up is not recommended for dogs with suspected musculoskeletal abnormalities. The Back-up should be used with caution in dogs with diagnosed lumbosacral pain, hip dysplasia or osteoarthritis, or cruciate ligament disease.

##### Progression

Progression for the Back-up can be broken down into several stages (Figures 8A–K). The first goal is to have the dog walk backwards a distance of 3 m (9.8 ft) 3 times in 30 s. The gait must be smooth and intentional, with each paw leaving the ground independently. Dragging of the paws or a kyphotic spine is incorrect form and the dog should not progress to the next level of difficulty. After walking backwards, the dog progresses to trotting backwards with the same criteria as the walk. After trotting backwards, the Back-up progresses to backing up stairs. Each step should be 15–30 cm (6–10 in) in height. The dog must be able to back up 18 steps (may be split into 3 trips up and down 6 steps) in 30 s. The criteria for progression to the next level is that all four limbs must independently clear each step. Simultaneous “hopping” of the hindlimbs over the stairs is improper execution of the exercise. Following backing up the stairs, the Back-up progresses to backing up a vertical wall into a forelimb only “handstand”.

##### Training

There are several ways to train the Back-up exercise. One method is to place the dog between two objects such as a wall and chair. The handler then steps in front of the dog with a lure in front of the dog's nose. The handler takes a step toward the dog and the dog will respond by stepping backwards. During initial training of the Back-up, the dog should be rewarded after each step backwards. Once the dog understands the movement, the reward frequency can be decreased. Moving toward the dog too quickly or placing the lure too low causes the dog to round its spine. Placing the lure too high causes the dog to sit. As a general principle for training the Back-up, a dog should take 2–3 steps backwards for every step forward by the handler.

### Foundational Fitness Assessment

#### Overview

The Foundational Fitness Assessment (FFA) objectively measures a dog's fitness across the foundational fitness components. We developed this assessment to aid in measuring a dog's current fitness, identifying any change in fitness after completing a training program, adjusting training programs, and comparing the fitness of dogs in similar age ranges, breeds, or careers. Measuring a dog's current fitness was the primary impetus for the development of this assessment. We wanted a way to objectively assess the fitness of the dogs in the PVWDC training program in order to establish a baseline before they began the foundational training. We also desired a formalized assessment to determine if the training program we developed was making a difference in a dog's fitness. This kind of assessment will now allow us to compare different training program styles, methods, durations, frequency, and equipment. We hypothesized that dogs would become more fit during the course of a training program, and a formalized assessment would allow us to adjust that training program to maximize their fitness development. Finally, we wanted to be able to objectively compare the fitness of dogs. This will eventually allow us to develop age, breed, and career-specific standards and scoring.

We prioritized durability, accuracy, and simplicity when we designed the FFA. In this setting, a durable assessment is one that is easily implemented in a non-professional setting, involves inexpensive and readily available equipment, and is quick to conduct. Accuracy amongst evaluators (inter-rater and intra-rater reliability) and between assessments (inter-assessment and intra-assessment) are also important. Finally, we wanted a simple test that either does not require new behaviors or utilizes behaviors that could be rapidly learned. Our end state is an assessment that could work for most dogs, by most evaluators, and in most environments.

As a result of our experience during the pilot implementation, the FFA is divided into two levels. Level One consists of two tests that evaluate core stability and whole-body power generation (predominantly hindlimb extension strength) without the requirement for learned behaviors. Level Two utilizes two behaviors that take ~4 weeks to learn but that provide an independent assessment of hindlimb extension strength and hindlimb stability. The two levels may be used separately or together, and future work will focus on validating their independent and consecutive use.

#### Foundational Fitness Assessment—Level One

##### Overview

The Foundational Fitness Assessment-Level One (FFA-L1) consists of the Sprint Test (ST) and the Progressive Plank Test (PPLT). This assessment requires minimal and inexpensive equipment and takes ~15–20 min per dog to complete. While the order of the FFA-L1 requires further exploration, we propose that the PPLT should be completed after the ST so that core stability fatigue does not affect the maximal sprint. Some dogs may require the PPLT before the ST for behavioral reasons. Regardless of the order, the dog should be given at least 5 min to recover between tests.

##### Sprint Test

The ST assesses a dog's ability to generate whole-body power during the acceleration phase (initial 25 m) of sprinting. Sprinting is predominantly a hindlimb extension movement (53), so the ST is primarily an assessment of hip, stifle, and tarsal extension strength. To perform the ST, an area of flat, level, and smooth ground (preferably grass, dirt, or turf) at least 50 m (164 ft) long and 10 m (33 ft) wide must be identified. As the dog will perform a maximal effort attempt, every effort to ensure the dog's safety must be taken.

The dog must start from a down position (chest and tarsi on the ground) with its entire body behind the starting line. Light restraint may be used to minimize the obedience requirements. Accurately measure the 25 m (82 ft) course and place a narrow but conspicuous marker (e.g., cone) to define the finish line. Any method may be used to motivate the dog to sprint maximally, but the motivation should be located at least 10 m beyond the finish line to encourage maximal effort for the duration of the test. A toy reward may be held by the handler and a “Come” or “Here” command given. A toy may be thrown beyond the finish line, but this method is not preferred as some dogs will decrease their effort to track the toy in flight. For a dog trained to a bite sleeve, a decoy running away from the finish line may be used.

Due to the need for precise measurement and the inherent margin of error with manual timing the ST must be recorded using a video camera capable of at least 30 frames per second (accurate to 0.033 s) and preferably 60 frames per second (accurate to 0.017 s) (54). The camera should be positioned in line with the finish line and out of the way of the dog's path. The camera is aimed at the starting line to capture the start and then rotated to capture the finish. The video is then analyzed to determine the interval (in hundredths of a second) between the first motion of the dog and the first portion of the body to cross the finish line or object. The dog is given three attempts with at least 2 min rest between attempts. The ST score is expressed as the seconds (to the hundredths place) of the fastest attempt (e.g., 3.08 s).

##### Progressive Plank Test

The PPLT assesses a dog's trunk muscle endurance in a safe and objective manner. The plank (or prone bridge) is reliably used to assess human core muscle endurance (10, 44, 47, 55–59). To perform the PPLT, the dog's Plank measurements are first obtained (see above). Next, the dog is given 45 s in which to accumulate 30 s of proper Plank position at Level 2. If the dog successfully completes this stage, they are given 30 s of rest. The dog is then given 45 s in which to accumulate 30 s of proper Plank position at Level 4. If the dog successfully completes this stage, they are given 30 s of rest, and the process is repeated for Level 6. If the dog successfully completes this stage, they are given 30 s of rest before attempting a final maximum duration Plank at Level 8. The dog is allowed 15 s of improper position during this final stage. The PPLT is terminated when the dog steps off either block, fails to accumulate 30 s of proper Plank position within 45 s during the Level 2, 4, or 6 stages, or accumulates 15 s of improper Plank position during the final Level 8 stage. The PPLT score is expressed as the final level and seconds (rounded down to the nearest second) completed at that level. Examples are 4–0:24 (24 s at Level 4) or 8–2:15 (2 min and 15 s at Level 8).

#### Foundational Fitness Assessment—Level Two

##### Overview

The Foundational Fitness Assessment-Level Two (FFA-L2) consists of the Progressive Pivot Test (PPT) and the Progressive Squat Test (PST). This assessment requires ~4–6 weeks of prior training, minimal and inexpensive equipment and takes ~15–20 min per dog. While the order of the FFA-L2 requires further exploration, we propose that the PPT should be conducted before the PST so that fatigue from the Squat does not affect hip stability. The dog should be given at least 5 min to recover after completion of the final PPT interval before beginning the PST.

##### Progressive Pivot Test

The PPT assesses a dog's hip stability in a safe, objective, and specific manner. To perform the PPT, the dog completes up to 3 complete Pivot rotations in under 30 s in both directions at successively higher levels until the dog's maximum level is reached. The dog has 45 s of total time in which to complete 30 s of active Pivot. The 30 s active time is paused if the dog steps off the object or if there is a handler or reward issue. The interval ends when the dog successfully completes the 3 rotations, the 30 s of active time elapses, or the 45 s of total time elapses. After completion of a level in one direction, the dog is given 30 s of rest before attempting that level in the opposite direction.

If the dog successfully completes the level, the object height is increased to the next level. If the dog successfully completes a level in one direction but does not complete the level in the other direction, the object height is not increased. This process is continued until either the dog fails to complete a level or the object height is raised to the dog's maximum level. Then, the dog completes a final 2 min maximum effort in each direction. The PPT score is expressed as the final level and number of rotations (rounded down to the nearest quarter rotation) in the clockwise (expressed first) and counterclockwise directions. Examples are 3–2.75/1.5 (two and three-quarter rotations clockwise and one and one-half rotations counterclockwise at Level 3) and M-10.25/12 (ten and one-quarter rotations clockwise and twelve rotations counterclockwise at this dog's maximum level).

##### Progressive Squat Test

The PST assesses a dog's hip, stifle, and tarsus extension strength and endurance in a safe, objective, and specific manner. To perform the PST, the dog completes up to 7 Squats in under 30 s at successively higher levels until the dog's maximum level is reached. The dog has 45 s of total time in which to complete 30 s of active Squats. The 30 s active time is paused if the dog steps off the object or platform or if there is a handler or reward issue. The interval ends when the dog successfully completes the 7 Squats, the 30 s of active time elapses, or the 45 s of total time elapses.

If the dog successfully completes the level, the object height is increased to the next level. After successful completion of a level, the dog is given 30 s of rest before attempting the next level. This process is continued until either the dog fails to complete a level or the object height is raised to the dog's maximum level. Then, the dog completes a final 2 min maximum effort. The PST score is expressed as the final level and number of Squats completed at that level. Examples include 2–5 (5 Squats at Level 2) and M-14 (14 Squats at this dog's maximum level).

#### Scoring and Standard Development

Our goal is to assess the foundational fitness of a sufficient number of dogs in order to develop both a scoring system and age, breed, and career-specific standards. We aim to develop a bell curve of results for each assessment. Then, we propose a scoring system where results clustered near the mean receive an average score while results above the mean receive higher scores and those below the mean receive lower scores. See Table 4 for more detail.

**Table 4:** Proposed Foundational Fitness Assessment scoring system.

**Proposed FFA scoring system**			
**Score**	**Description**	**Percentile range**	**Percentage of results (%)**
0	At risk	0th−10th	10
1	Minimal	11th−36th	25
2	Effective	37th−63rd	26
3	Excellent	64th−89th	25
4	Outstanding	90th−100th	10

We also propose further work to explore the relationship between foundational fitness results, injury, and objective career-specific performance measures. We are interested in identifying the effect a dog's fitness has on the likelihood it will experience an injury, the type of injury (acute or degenerative), the severity of injury, and the duration of recovery. We hypothesize that more fit dogs will be injured less frequently, experience fewer degenerative injuries, be injured less severely, and recover faster than their less fit counterparts. We also believe a dog's fitness is integral to its ability to perform its job or sport. While performance in some dog activities is easy to measure (e.g., distance in dock diving or time in agility), other dog activities are harder to measure or have more poorly defined performance metrics (e.g., explosive detection or urban search and rescue). We hypothesize that more fit dogs will perform better at their activities whether those activities are predominantly physical or less physically-focused.

## Results

Our aim was to develop a formalized method to develop and assess foundational fitness in working dogs. We implemented the FTW program in the PVWDC population over an ~3-month period consisting of training personnel, familiarizing dogs, initial assessment, and regular training. Our pilot results are summarized below.

### Safety

The FTW program was safe in this group of dogs, under the conditions tested. We assessed 31 dogs on two occasions without injury or negative effect on training. The same group of dogs conducted ~600 foundational training sessions during the familiarization and regular training periods. We identified several minor abrasions from contact with the concrete blocks but no other injuries. The formalized Cool-down routine with its paw, pad, and nail check conducted after every training session facilitated identification and treatment of injuries before the dog left for the day. Without this system, injuries sustained during FTW or other training could have been overlooked or identified at home by a non-professional foster without medical resources.

The PVWDC veterinarians also experienced the common phenomenon in human athletic performance where the athletic trainer identifies an athlete's musculoskeletal issues before a medical professional does. On numerous occasions we had a non-trainer individual conduct FTW and then bring us a data-driven issue. Comments such as “I was just doing Pivot with Ivey, and both times she did 2–2.5 rotations clockwise but only 1–1.5 rotations counterclockwise. Can you come take a look?” resulted in a targeted musculoskeletal examination, identification of the issue (unilateral quadriceps femoris muscle soreness in this case), treatment, and rapid return to full performance.

Finally, we believe FTW to be safe for young dogs. The primary concerns with fitness training in these dogs are the effects of excessive force on epiphyseal plates and repetitive motion on the skeleton and joints (60–63). The resistance focus, short duration, and low-impact nature of the foundational fitness exercises and assessments is not likely to cause musculoskeletal damage, although monitoring is needed. In addition, regular joint loading in a structured and progressive manner in young dogs may decrease the risk of musculoskeletal damage (64–68).

### Ease of Implementation

FTW appears to be an accessible method to develop and assess fitness in working dogs. Dogs of diverse breeds, temperaments, and ranging in age from 2 months to 3 years learned to perform each of the movements. Dogs were also appropriately challenged by the program. After initial familiarization, dogs who tested well on the Plank were then advanced to higher levels while dogs who scored lower on the Plank were provided lower progressions appropriate for them.

Puppies started learning proprioception and height-adjusted hip stability during their first week at the PVWDC, and dogs that had trained fitness for years in our program adjusted as well. We found that dogs that started FTW earlier (before 6 months of age), were exposed to the exercises more frequently, and practiced the exercises outside of the foundational training sessions rapidly learned the exercises and progressed. While dogs over 6 months of age and dogs that were only exposed to the exercises during the foundational training sessions took longer to learn the exercises, the limited number of exercises, repetition, and formalized criteria and progressions assisted their learning.

FTW is also an accessible program for the people involved in assessing and developing working dog fitness. The feedback from trainers, interns, and volunteers on the foundational fitness training program centered on simplicity and ease of progression. Our personnel found the reduced number of movements and formalized structure simple to implement. The clarified criterion and standardized levels made determining when and how to progress the difficulty of a movement easy.

The trainers, interns, and volunteers universally adopted the revised FTW program. The feedback we received identified an increase in perceived relevance to a working dog's future career as a major factor in this adoption. Also, the significantly reduced training requirements increased the confidence of trainers with less marker (e.g., clicker) training experience. We consistently observed trainers taking 2–3 weeks to become comfortable enough to teach an intern who then took the same length of time before teaching a volunteer. It took ~4–6 weeks for the FTW knowledge to pass through four generations (FTW expert to trainer to intern to volunteer) and make a novice competent to perform the training.

## Discussion

We developed a formalized method to train and assess foundational fitness modalities for working dogs. The PVWDC FTW program incorporates posture development and frequent reinforcement of this behavior, warm-up and cool-down routines to prepare for and recover from training, methods to efficiently train foundational fitness, and a two-level format for assessing foundational fitness. We implemented this program in a working dog training facility and demonstrated the program's safety and ease of implementation.

Some limitations to this initial implementation are the young age of some of the dogs in our population, the prior exposure of our dogs and personnel to fitness training, the unique structure of the PVWDC, and the short duration of the implementation period. The young age of some of the dogs in our program allowed us to determine optimal methods for early familiarization with fitness exercises but limited our ability to see how more mature dogs would fare. In addition, PVWDC has a culture of fitness, and our personnel were accustomed to performing fitness training with our dogs. These factors likely shortened the learning curve for both our dogs and our personnel. While not completely unique among working dog programs, our pattern of bringing dogs into our program at 8 weeks of age and training with them on a daily basis until 12–24 months of age provided more and earlier contact time than some programs. Finally, the formalized and quantitative methods described in this paper represent an implementation period of ~4 months. The number of refinements accomplished in that time indicate more are likely as we continue to train working dog fitness.

The aim of this paper was to initially describe these pilot techniques to assess and train foundational fitness for working dogs. Thus, further prospective studies are needed to validate the assessment components and training exercises. Also, this initial implementation allowed us to define initial progression levels for some exercises (Pivot, Plank, and Squat). The progression for other exercises (Posture Down, Chipmunk, and Back-up) is more generally described. Further work remains to be done to define the optimal method, sequence, and rate to progress each exercise.

Formalized canine fitness programs are rare. Even fewer are suitable for the functional fitness requirements and temperaments of working dogs or the time and logistical constraints of a kennel or training facility. In contrast, the human fitness realm has numerous methods to assess and train fitness for tactical athletes in comparable organizations (8, 11, 69–73). In addition, throughout this article we have cited research into the effectiveness of various human fitness exercises and methods; working dogs need similar evidence-based methods to assess and train fitness. We believe the PVWDC FTW program is one step in that direction.

We anticipate this work will serve to add momentum to the growing field of canine performance medicine. One gap deserving future research is the quantification of muscle activity for various fitness exercises. While these investigations have been started with rehabilitation movements (49), much work remains to be done to understand which exercises most effectively activate the desired musculature. Another area for future research is developing alternate exercises and progressions to develop foundational fitness. While we believe exercises like the Squat and Pivot are cornerstones of fitness, a dog's continued progression depends on incorporating alternate methods to develop the same fitness modalities.

In addition to the exercises themselves, we believe research needs to be done to provide evidence for the optimum programming and periodization methods. For training sessions, the frequency, timing throughout the day, and timing relative to work or other training need to be explored. The proper order of exercises within the session along with the optimum combination of sets, repetitions, and intensities needs to be determined. The canine fitness realm also needs safe and effective methods for developing strength and power similar to the barbell and kettlebell for human fitness.

Finally, formalized canine fitness must grow beyond these foundational fitness roots. Various working and sporting dog careers and disciplines have specific fitness requirements that should be layered on top of foundational fitness. Dogs need speed, power, endurance, and agility to perform in these careers, and their handlers and trainers need evidence-based training methods and assessments to help them improve those modalities.

We believe multidisciplinary collaboration is the key to unlocking progress toward filling these gaps. Working dog handlers, experienced trainers, and canine performance-oriented veterinarians should partner with a diverse array of similarly-oriented scientists to solve these challenges. The PVWDC is eager to collaborate with like-minded individuals, kennels, programs, and organizations.

## Ethics Statement

The animal study was reviewed and approved by the University of Pennsylvania Institutional Animal Care and Use Committee.

## Author Contributions

BF and MR developed the method, conducted the pilot implementation, and participated in manuscript preparation. CO developed the method and participated in manuscript preparation. All authors contributed to the article and approved the submitted version.

## Conflict of Interest

The authors declare that the research was conducted in the absence of any commercial or financial relationships that could be construed as a potential conflict of interest. The reviewer LW declared a past co-authorship with one of the authors CO to the handling editor.
